# RESEARCH CHALLENGES IN STAGE III AND IV RAS-ASSOCIATED CANCERS: A Narrative Review of the Complexities and Functions of the Family of *RAS* Genes and Ras Proteins in Housekeeping and Tumorigenesis

**DOI:** 10.3390/biology14080936

**Published:** 2025-07-25

**Authors:** Richard A. McDonald, Armando Varela-Ramirez, Amanda K. Ashley

**Affiliations:** 1 Global Health Science Institute, Las Cruces, NM 88001, USA; 2 The Border Biomedical Research Center (BBRC), Department of Biological Sciences, The University of Texas at El Paso, El Paso, TX 79968, USA; avarela2@utep.edu; 3 Department of Chemistry and Biochemistry, New Mexico State University, Las Cruces, NM 88003, USA; ashleyak@nmsu.edu

**Keywords:** animal models, late-stage cancer, oncogenic signaling, *RAS* genes, Ras proteins, tumorigenesis, stage III cancer, stage IV cancer, tumor microenvironment

## Abstract

Proto-oncogenes in the *RAS* superfamily play dual roles in maintaining cellular homeostasis, such as regulating growth signals and contributing to cancer development through proliferation and deregulation. Activating proto-oncogenes in vitro transforms cells, underscoring their centrality in gene regulation and cellular networks. Despite decades of research, poor outcomes in advanced cancers reveal gaps in understanding Ras-driven mechanisms or therapeutic strategies. This narrative review examines *RAS* genes and Ras proteins in both housekeeping functions, such as cell growth, apoptosis, and protein trafficking, as well as in tumorigenesis, integrating insights from human (*HRAS*, *KRAS*, *NRAS*), mouse (*Hras*, *Kras*, *Nras*), and *Drosophila melanogaster* (*ras*) models. While *RAS* mutations are tightly linked to human tumors, the interplay between their standard and oncogenic functions remains complex. Even within the same tissue, distinct cancer pathways—such as the mitogen-activated protein kinase (MAPK) and phosphoinositide 3-kinase (PI3K) pathways—can drive varied disease courses, complicating treatment. Advanced-stage cancers add further challenges, including heterogeneity, protective microenvironments, drug resistance, and adaptive progression. This synthesis organizes current knowledge of RAS gene regulation and Ras protein function from genomic alterations and intracellular signaling to membrane dynamics and extracellular interactions, offering a layered perspective on the Ras pathway’s role in both housekeeping and tumorigenic contexts.

## Simple Summary

Cancers involving *RAS* gene mutations are among the most difficult to treat, particularly in advanced stages. These genes for *RAS*—*KRAS*, *HRAS*, and *NRAS*—control normal cellular processes, but when mutated, they help tumors grow, spread, and resist treatment. Decades of research have targeted *RAS* genes and Ras proteins, yet many therapies still fail due to oversimplified signaling interpretations and poor translational reliability. This review compares how Ras functions in healthy and cancerous cells and evaluates model systems across human, mice, and fly studies. We highlight the limitations of current model systems and the need for more physiologically relevant research tools. The goal is to push research toward more effective tools, refined models, and improved outcomes for patients with late-stage *RAS*-driven cancers.

## 1. RAS Introduction

Despite a global investment of over $250 billion in curative cancer treatments, survival rates for Stage III and IV human cancers (*late-stage cancers*) have remained essentially unchanged since 1930 [1]. This therapeutic gap is especially pronounced in cancers driven by mutations in *RAS* genes and their associated Ras proteins, including melanoma, leukemia, thyroid, lung, kidney, liver, prostate, pancreatic, and colorectal cancers [2,3,4,5]. This narrative review focuses on the *RAS* genes (*HRAS*, *KRAS*, *NRAS*) and Ras proteins (Hras, Kras, Nras) in humans; the *RAS* genes (*Hras*, *Kras*, *Nras*) and Ras proteins (Hras, Kras, Nras) in mice; and the *ras* gene and Ras protein in *Drosophila melanogaster* (hereafter referred to as *Drosophila*). To orient the reader, this review adopts an “inside-out” structure—beginning with intracellular and nuclear functions of RAS genes and Ras proteins, progressing to their membrane-associated roles, and ultimately examining how these functions extend into extracellular and microenvironmental interactions.

For clarity, the terminology uses *HRAS*, *KRAS*, and *NRAS* for human genes and Hras, Kras, and Nras for mouse genes, and *ras* for *Drosophila*. *RAS* gene mutations account for ~20% of all human cancers, corresponding to around 260,000 new cases in the United States each year [6]. Box 1 summarizes key terms, and Table 1 highlights cancer types associated with *KRAS* mutations, underscoring the urgent need for targeted therapies. *RAS*-mutated cancers typically carry poor prognoses under standard chemotherapy, highlighting the urgent need for alternative strategies [7]. Kirsten *RAS*, or *KRAS*, gene mutations are found in approximately 90% of pancreatic ductal adenocarcinomas (PADC) [8,9,10,11], which account for about 90% of all PADC [12,13], and in 30–35% of lung adenocarcinomas, a primary subtype of lung cancer [14,15,16,17,18].

Box 1 **Key abbreviations used in this review.**

*RAS* and Ras—rat sarcoma [virus] gene and protein, respectively, refer generically to the Ras family.

*HRAS* and HRas or H-Ras—human species: Harvey rat sarcoma [virus] gene and protein, respectively.

*KRAS* and KRas or K-Ras—human species: Kirsten rat sarcoma [virus] gene and protein, respectively.

*NRAS* and NRas or N-Ras—human species: neuroblastoma rat sarcoma [virus] gene and protein, respectively.

*Hras* and Hras or H-ras—mouse species: Harvey rat sarcoma [virus] gene and protein, respectively.

*Kras* and Kras or K-ras—mouse species: Kirsten rat sarcoma [virus] gene and protein, respectively.

*Nras* and Nras or N-ras—mouse species: neuroblastoma rat sarcoma [virus] gene and protein, respectively.

*ras* and Ras—*Drosophila* species: rat sarcoma [virus] gene and protein, respectively.

A: *HRAS*, *KRAS*, *NRAS* and H-Ras, K-Ras, N-Ras—gene and protein, respectively, when species unspecified.

B: The use of “rat” in naming these genes and proteins is due to their initial discovery in rat models.

Rap-1—Ras-proximate-1 or RRSP Ras-related protein 1; RASAL2—Ras protein activator like 2.

Ras, Rho, Rab, Arf, Ran—Ras sarcoma virus, Ras homologous, Ras-related protein in the brain, ADP-ribosylation factor, Ras-related nuclear protein. Noted as a group based on the discovery timeline.

AKT—protein kinase B; ATP—adenosine triphosphate.

CAAX motif—C-terminal tetrapeptide with a cysteine (C), two aliphatic amino acids (A1 and A2), and a variable terminal amino acid position (X).

cAMP—cyclic adenosine monophosphate.

EMF—electromagnetic field.

G protein—guanine nucleotide-binding protein.

GAPs—GTPase activating proteins; GATA2—guanine-adenine-thymine-adenine-binding factor 2; GDI—guanosine dissociation inhibitor; GEFs—guanine nucleotide exchange factors.

Gln^61^ or Q61—glutamine at position 61; Gly^12^ or G12—glycine at 12; Gly^13^ or G13—glycine at 13.

GTP—guanosine triphosphate; GTPase—guanosine triphosphatase.

HBV—hepatitis B virus; HIV-1—human immunodeficiency virus type-1.

HIF-1—heterodimeric transcription factor (HIF-1α/β); not interchangeable with gene symbol HIF1A.

HVR—hypervariable region.

KRas^G12C^—an oncogenic driver glycine to cysteine mutation at residue 12.

MAPK—mitogen-activated protein kinase; MAPK/ERK—MAPK/extracellular signal-regulated kinase.

MRTX1133—KRas^G12C^ inhibitor, also known as Adagrasib.

MyD88—myeloid differentiation factor 88.

NSCLC—newly diagnosed non–non-small-cell lung cancer.

PI3K—phosphoinositide 3-Kinase; PI3K-PKB/AKT—phosphoinositide 3-kinase/protein kinase B, also known as AKT; PIK3CA/AKT—phosphatidylinositol 4,5-bisphosphate 3-kinase catalytic subunit alpha/AKT

PD-1 receptor—programmed cell death protein 1 receptor or CD279.

PEBP1, PEBP2, PEBP3—phosphatidylethanolamine-binding protein 1, protein 2, protein 3, respectively.

PGA2—prostaglandin A2; PGF2α—prostaglandin F2 alpha; PGI2—prostacyclin.

RGBARG—a multidomain protein containing: regulator of chromosome condensation 1 [RCC1], Rho guanine nucleotide exchange factor [RhoGEF], Bin/Amphiphysin/Rvs domain [BAR], Ras GTPase-activating protein [RasGAP].

Ras/Raf/MEK/ERK—rat sarcoma virus, rapidly accelerated fibrosarcoma, mitogen-activated protein kinase-extracellular signal-regulated kinase.

Table 1 presents several cancers where a high percentage of patients carry *KRAS* gene mutations. These mutations are critical drivers of tumor progression in pancreatic, colon, and lung adenocarcinomas, influencing key cellular pathways. Understanding the prevalence and role of *KRAS* gene mutations is crucial for unraveling the genetic basis of these cancers and developing targeted therapies that effectively exploit these mutations.

Advanced-stage cancers face pharmacokinetic and pharmacodynamic challenges, including poor drug delivery and significant toxicity from high-dose therapies [19,20,21]. Distinct tumor regions carry different mutations, creating adaptive resistance to targeted treatments [20,21]. The tumor microenvironment, comprising stromal cells, immune cells, and extracellular matrix components, often shields cancer cells by providing protective niches and promoting immune evasion [22,23,24,25,26]. Dynamic polyclonal evolution and metastasis further complicate treatment [27,28,29,30]. The limited efficacy of current targets, such as the varying responses of different human *KRAS* gene mutations to specific inhibitors, such as Sotorasib for KRAS^G12C^, Adagrasib for KRAS^G12C^ [31,32,33], underscores the need for precise elucidation of mutations and their impact on cellular processes driving tumorigenesis, including oncogene addiction, mutation variants [34,35,36,37].

Adding to these challenges is the significant limitation of animal models, particularly mice, in translating findings into effective human therapies, especially for late-stage cancers [38,39]. Simplified genetics, metabolic discrepancies, and differences in the tumor microenvironment result in drugs that appear promising in animal studies but fail in human trials, highlighting the need for a “bridge” between animal models and human applications [40]. Advanced human cancers are highly heterogeneous, characterized by diverse genetic and epigenetic alterations, including complex *RAS* gene mutations, such as co-occurring mutations, structural variants, or regulatory disruptions, which drive aggressive disease progression over extended periods. In contrast, mouse models often rely on the rapid induction of cancer using genetic engineering or chemical carcinogens, which fail to mimic the gradual progression and metabolic adaptations observed in human tumors. For example, human tumors are presumed to exploit the Warburg effect for energy production [41], but mice’s faster basal metabolic rates can overestimate the efficacy of glycolysis-targeting treatments. Similarly, drugs like paclitaxel are metabolized and cleared more rapidly in mice, potentially underestimating the toxicities observed in humans. These metabolic discrepancies further limit the translational applicability of findings from mouse models, driving the ongoing search for alternative or complementary models [42].

Moreover, the tumor microenvironment in late-stage human cancers involves intricate interactions between cancer cells, immune cells, endothelial cells, and surrounding tissues, which differ significantly from those in animals [43,44,45,46,47]. Metastatic tumors in humans, for example, colonize specific niches in organs like the liver, lungs, or bones, which animal models fail to replicate accurately. This discrepancy often leads to misleading therapeutic responses in animal studies that do not effectively translate to humans. Additionally, immunotherapies, crucial in treating advanced cancers, rely heavily on the interaction between the tumor and the immune system. However, the immune systems of animal models, particularly mice, differ substantially from those of humans. Therapies promised to reduce tumor size or metastasis in animals often fail to induce the same immune-mediated reactions in humans, leading to unsuccessful clinical trials for late-stage cancers [43,48]. Metastasis, a hallmark of late-stage cancer, is also poorly replicated in animal models. Although animals can be genetically engineered to develop tumors, metastasis progression in specific human organs involves unique biological and environmental factors that are not faithfully reproduced in animal models, making it difficult to predict drug efficacy in metastatic human cancers based solely on animal studies [49,50]. These model limitations are particularly concerning in studying RAS-driven cancers, where complex signaling dynamics and microenvironment feedback loops, both central to RAS function, are often misrepresented in animal studies.

In addition, the signaling interactions of Ras proteins among housekeeping roles such as cell growth regulation, apoptosis, and intracellular protein trafficking, tumor cells and their host environment remain incompletely characterized in the context of late-stage cancers. Building on the translational limitations described above, this analysis avoids emphasizing failed therapeutic strategies. Instead, it centers on the underlying biological roles of *RAS* genes, their proteins, and orthologs across humans, mice, and *Drosophila*. A brief introduction to the RAS superfamily will also be provided. The analysis progresses from their intracellular roles in cellular maintenance and tumorigenesis to their interactions outside the cell membrane, including those with microbial elements. The following section examines the genetic and protein-level functions of Ras proteins, exploring how they contribute to cellular homeostasis, interact with the tumor microenvironment, and ultimately influence cancer progression. Clarifying these roles, especially in late-stage cancers, is critical for understanding *RAS* gene mutations, their regulatory functions, and their relevance to developing more effective therapeutic strategies.

Ras proteins function as central intracellular signaling mediators, cycling between inactive GDP-bound and active GTP-bound states [51]. Upstream regulators, such as receptor tyrosine kinases, guanine nucleotide exchange factors (GEFs), and adaptor proteins like growth factor receptor-bound protein 2, facilitate the activation of Ras proteins in response to external stimuli, including growth factors, cytokines, and hormones [52]. Once activated, GEFs promote the exchange of GDP for GTP, enabling Ras proteins to adopt an active conformation capable of engaging downstream effectors [53]. Downstream, Ras activation triggers signaling cascades, including the mitogen-activated protein kinase (MAPK) and phosphoinositide 3-kinase (PI3K) pathways, which govern cell proliferation, differentiation, and apoptosis [54]. Dysregulation in these interactions, whether caused by genetic mutations or disrupted upstream and downstream controls, can drive oncogenesis and influence the tumor microenvironment.

The role of *RAS* genes in the multi-step process of carcinogenesis remains incompletely understood, particularly regarding the specific stages at which *RAS* activation contributes to tumorigenesis and how the activated Ras proteins drive cell transformation. The discussion evaluates the biochemical and biological roles of *RAS* genes and *Ras* proteins, emphasizing their functions in housekeeping processes and tumorigenesis. Extending the focus beyond intracellular activities, it also briefly explores microbial interactions and the potential role of retroviral exposure in activating proto-oncogenes. By tracing this continuum, from proto-oncogene to oncogene, this evaluation provides insights into the impact of *RAS* gene mutations, their Ras proteins, and how they contribute to cancer progression.

Many RAS studies, particularly those from the late 20th century, defaulted to mouse nomenclature (*Hras*, *Kras*) regardless of the model organism used. This practice creates ambiguity when interpreting cross-species findings. In recent years, improved adherence to nomenclature conventions has clarified species-specific insights, though legacy inconsistencies persist. In this manuscript, we adhere to species-specific nomenclature, explicitly distinguishing human (*HRAS*, *KRAS*, *NRAS*), mouse (*Hras*, *Kras*, *Nras*), and *Drosophila* (*ras*) genes, unless referring to general *RAS* gene or Ras protein mechanisms not tied to a specific model.

Although this review focuses on the classical *RAS* gene family, including *HRAS*, *KRAS*, and *NRAS*, some genomic databases, including cBioPortal, extend *RAS* signaling classifications to include Ras-like genes like *RIT1*, which can be confusing. *RIT1* is part of the Ras superfamily and contributes to the MAPK pathway. Still, it lacks defining features of the canonical *RAS* proto-oncogenes, including the CAAX motif required for membrane targeting, and follows a distinct evolutionary trajectory. While *RIT1* mutations are implicated in cancers like lung adenocarcinoma and myeloid malignancies, they do not constitute canonical *RAS* mutations. This review excludes *RIT1* from the core RAS gene classification to maintain mechanistic clarity.

Also, for clarity, in this manuscript—as noted in Box 1, and except in subtitles—*RAS* refers to the human gene (italicized, all caps) and is thereafter cited simply as *RAS*, not “*RAS* gene”. Similarly, Ras refers to the human protein (non-italicized, mixed case) and is cited as Ras, not “Ras protein”. This convention applies to mouse and *Drosophila* models, following species-specific capitalization and formatting. Lastly, *RAS* or Ras may be used in singular and plural forms without modifiers such as “protein” or “proteins”.

The following section introduces the Ras superfamily, an evolutionarily conserved group of small GTPases that regulate diverse cellular functions in multiple species, to contextualize RAS within a broader biological framework.

## 2. RAS Superfamily: Evolutionary Conservation and Functional Diversification Across Species

Ras superfamily is a conserved group of small GTPases that regulate signal transduction, cytoskeletal organization, vesicle trafficking, and nucleocytoplasmic transport in diverse species [51,55]. Divided into five core families: Ras, Rho, Rab, Arf, and Ran, these proteins act as molecular switches, cycling between active GTP-bound and inactive GDP-bound states to precisely control key cellular pathways [56,57,58,59]. Conventionally, families are listed in the order of their discovery: Ras, Rho, Rab, Arf, and Ran [60]. This section summarizes their conserved roles and relevance to disease research in humans, mice, and *Drosophila*, setting the stage for a focused analysis of *RAS* and its downstream regulation.

Ras Family Overview: In humans, Ras, encoded by HRas, KRas, and NRas, regulates key oncogenic signaling cascades such as the MAPK and PI3K pathways, driving cell proliferation, differentiation, and survival [55,56,60]. Mouse models show that Kras knockout is embryonic lethal, while Hras and Nras are partially redundant [55]. In *Drosophila*, the homolog Ras85D controls photoreceptor development, oogenesis, and embryogenesis, making it a powerful genetic tool for dissecting conserved Ras-driven signaling pathways [55,61].

Rho Family Overview: Human Rho GTPases, including RhoA, Rac1, and Cdc42, orchestrate cytoskeletal dynamics, stress fiber formation, cell polarity, and migration, processes critical for cancer invasion and metastasis [55,60]. In mice, RhoA is essential for smooth muscle function, Rac1 regulates neuronal migration, and Cdc42 governs immune cell polarity and axonal development [55,60]. *Drosophila* Rho1, Rac1, and Cdc42 regulate epithelial morphogenesis, cytoskeletal organization, and oocyte polarity, underscoring their evolutionary conservation [55,61].

Rab Family Overview: Human Rab proteins, specifically Rab5, Rab7, and Rab11, regulate vesicle trafficking, lysosomal targeting, and endosomal recycling, thereby maintaining membrane dynamics and cellular homeostasis [55,60]. In mice, Rab5 and Rab7 are essential for neuronal trafficking, while Rab11 is required for epithelial polarity and lumen formation [55,60]. In *Drosophila*, loss-of-function mutations in Rab5, Rab7, and Rab11 disrupt endocytic transport, lysosomal function, and epithelial recycling, highlighting their conserved roles across species [55,61].

ARF Family Overview: Human Arf1 and Arf6 localize to the Golgi and plasma membrane, mediating vesicle budding, membrane trafficking, and cytoskeletal remodeling [55,60]. Mouse Arf1 is required for Golgi integrity and embryogenesis, while Arf6 regulates endocytosis and membrane organization [55,60]. *Drosophila* Arf1 and Arf6 maintain Golgi structure and epithelial trafficking during development, reflecting strong functional conservation [55,61].

Ran Family Overview: Human Ran coordinates nucleocytoplasmic transport and mitotic spindle assembly, supporting nuclear organization and cell division [55,57]. In mice, Ran is essential for embryonic cell cycle progression, as evidenced by the mitotic arrest caused by knockout [55,60]. *Drosophila* Ran controls spindle formation during oogenesis and early embryonic development, emphasizing its conserved role in cell cycle regulation [55,61].

The evolutionary conservation of the superfamily makes it a valuable framework for understanding disease mechanisms [57,58,59]. Building on this cross-species framework, the following section examines the genetic architecture, regulatory mechanisms, and functional consequences of *RAS* and its regulation at the protein level.

### RAS Gene Mutations and Ras Protein Regulation

Identified in the 1960s, the Harvey rat sarcoma (*Hras*) and Kirsten rat sarcoma (*Kras*) viral oncogenes were among the first cell-transforming genes discovered in rats [62,63]. These genes were activated initially through retroviral mechanisms: specific retroviruses captured the host rat genes *Hras* and *Kras*, incorporating them into their viral genomes. This transduction process produced the viral oncogenes *HRAS* and *KRAS*, which were later shown, along with *NRAS*, to play key roles in human cancer [64]. These oncogenic genes drive uncontrolled cellular proliferation.

Although first studied in retroviral models, homologous genes were eventually found in humans. Human *HRAS*, *KRAS*, and *NRAS* encode proteins that normally regulate cell growth and differentiation, but mutations in these genes disrupt this control. Approximately 94% of human pancreatic ductal adenocarcinoma (PDAC) harbor *KRAS* mutations, 86% of which involve a missense substitution at glycine 12 (G12) [5]. The *KRAS* G12 missense mutation is a major driver of pancreatic tumor development [8]. Similarly, *KRAS* mutations occur in 35% to 45% of colorectal cancers [16]. *KRAS* activation in colorectal cancer is associated with the upregulation of downstream genes such as *BCL2*, *H2AFZ*, *RAP1B*, *TBX19*, *E2F4*, and *MMP1*, many of which are being investigated as markers of clinical progression [65].

The human genes *HRAS, KRAS, and NRAS* contain seven exons, three of which are part of the binding region for the Spl housekeeping proteins [66]. In contrast, sequence-specific DNA-binding proteins are confined to the promoter region of the *HRAS, KRAS, and NRAS* [67]. The human proteins HRas, KRas, and NRas are 21 kDa monomeric guanine nucleotide-binding proteins (GTPases) with similar structures and functions. However, they differ in tissue-specific expression patterns, mutation prevalence in cancers, and interactions with cellular signaling pathways, contributing to their distinct roles in oncogenesis [53].

**Figure 1:**
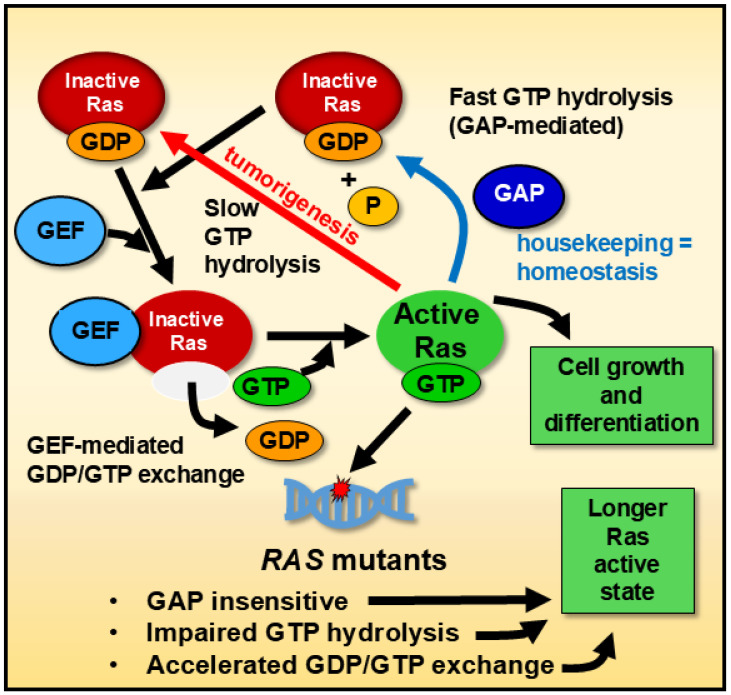
General mechanism of Ras GTPase regulation. Ras cycles between an inactive guanosine diphosphate (GDP)-bound state and an active guanosine triphosphate (GTP)-bound state. Two types of regulatory enzymes control this cycling: guanine nucleotide exchange factors (GEFs) and GTPase-activating proteins (GAPs). GEFs promote the exchange of GDP for GTP, driving Ras into its active GTP-bound state. In contrast, GAPs accelerate the hydrolysis of GTP to GDP (GAP-stimulated = fast hydrolysis), thereby returning Ras to its inactive state. Mutations in Ras can impair GTP hydrolysis, causing the protein to remain active for extended periods compared to wild-type Ras. This model applies broadly to human, mouse, and *Drosophila* systems, where Ras GTPase activity follows conserved regulatory mechanisms. Dysregulated Ras signaling is a hallmark of many advanced cancers, including those in late-stage tumors, where persistent Ras activation contributes to tumor progression, metastasis, and resistance to therapies.

*RAS*, members of the proto-oncogene family that encodes guanyl-nucleotide-binding proteins, are of significant interest due to their potential implications in human cancer treatment. The mutated forms of HRas, KRas, and NRas, when bound to guanosine triphosphate (GTP), remain persistently activated, disrupting normal regulatory mechanisms [68]. Generally, GTPase hydrolytic activity is slow, but this activity is rapid in the presence of GTPase-activating proteins (GAPs). Furthermore, Ras proteins have intrinsic GTPase activity [69]. However, given the complex interplay between housekeeping and tumorigenesis processes, even after decades of research, the well-understood slow/fast hydrolysis mechanisms have yet to be translated into beneficial anticancer treatments in humans. As with other G proteins, while Ras’ GTP-bound form is active, Ras’s GDP-bound form is inactive (Figure 1). A single nucleotide point mutation in the human *RAS* can result in a single amino acid substitution in Ras, altering its GTPase activity and downstream signaling [70]. In some instances, the mutated Ras exhibit a more active function in non-cancerous cells, promoting accelerated cell proliferation and differentiation, which favors the origin of cancer cells compared to the non-mutated ones [70]. To maintain homeostasis, Ras is typically activated by GDP/GTP interchange facilitated by guanine nucleotide exchange factors (GEFs) and is inactivated by GTP hydrolysis induced by GAPs [71].

These oncogenic Ras mutants evade inactivation through various mechanisms. For instance, they cannot efficiently hydrolyze GTP, prolonging their active, GTP-bound state, though recent work has demonstrated pharmacological restoration of GTP hydrolysis in some mutant RAS isoforms [72]. These oncogenic Ras mutants evade inactivation through various mechanisms [73]. For instance, they cannot efficiently hydrolyze GTP, prolonging their active, GTP-bound state [74]. Moreover, Ras mutants are insensitive to GAPs and exploit accelerated GDP/GTP exchange, further extending their active state [75].

Most human and mouse Ras undergo post-translational polyisoprenylation at the C-terminal cysteine 186, facilitated by an acylation reaction. For example, while Ras requires additional palmitoleate modification at amino acid 181 for plasma membrane anchoring, Kirsten rat sarcoma viral oncogene homolog 4B does not rely on this step; instead, it utilizes a polybasic domain for membrane association [76,77,78]. Methylation and proteolysis are essential for the efficient membrane binding of the prenylated Ras, as demonstrated in studies focusing on human KRAS4B [79]. A thioester linkage between palmitic acid and a cysteine residue near the carboxyl terminus further anchors Ras to the plasma membrane. Membrane anchoring mechanisms vary among Ras: palmitoylation of one or two cysteine residues anchors human and mouse Ras isoforms by a sequence of lysine residues in its polybasic domain [80]. Subcellular membrane localization determines the discrete signaling activities of Ras [81]. Biological differences among Ras isoforms can be attributed to their specific subcellular localization, directing their distinct functions [81]. Consequently, whether mutated or unmutated, proteins from Ras activate different trafficking and metabolic pathways depending on their endomembrane microenvironments. The CAAX motif (C-terminal tetrapeptide consisting of a cysteine [C], two aliphatic amino acids [A1 and A2], and a variable terminal amino acid position [X]) targets Ras to the endomembrane, where it undergoes post-translational modifications necessary for efficient membrane association [76]. Specifically, NRas and HRas are transiently localized in the Golgi before reaching the plasma membrane, whereas KRas exhibit less Golgi localization [82]. Although hamster-derived cells were used in this study, the authors employed nomenclature typically associated with mouse genes and proteins. This practice reflects the historical nomenclature for gene and protein, denoted here as *RAS*/Ras for the gene and the protein, respectively, which aligns with research conventions but introduces species-specific ambiguity. These post-translational modifications are crucial for the proper trafficking and activity of Ras. Additionally, palmitoylated Ras traffics through recycling endosomes to the plasma membrane during exocytosis, highlighting the importance of endomembrane localization in their function [83].

The overall similarities among Ras from related gene products and different species include more than 95% homology for amino acids 1 to 80, 70% to 80% homology for amino acids 81 to 160, a hypervariable region for amino acids 161 to 185, and a highly conserved region for amino acids 186 to 189 [53,84,85,86]. This conserved region contains the amino acid sequence of cysteine, valine, lysine, and serine, all of which are aliphatic [87,88]. In vitro mutagenesis on amino acids 116 to 119 and 143 to 147 directly affects GTP binding [89,90]. The asparagine at position 116 forms hydrogen bonds with the O-6 residue of guanine, whereas the aspartate at position 119 forms hydrogen bonds with the two amine groups of guanine nucleotide. Glutamine (Gln) at position 143 and lysine at position 147 interact with the purine ring of guanine [91]. In contrast, the amino acids tyrosine at position 32 and tyrosine at position 40 have GAP binding activity. The amino acid residues 5 to 63, 77 to 92, 109 to 123, 139 to 165, and 186 to 189 are essential for Ras activity [84,92]. Consequently, these amino acid residues are classified as the essential protein domain. Amino acid residues 32 to 40, which reduce the biological effect of Ras, interact with downstream effectors and regulatory proteins and are categorized as part of the effector domain [84,93].

Highlighting how *RAS* functions integrate into broader cellular networks and downstream pathways (beyond signaling alone) can give valuable insights into its role in normal and malignant processes. This system-oriented perspective encourages viewing *RAS* as part of dynamic, multi-tiered feedback networks that influence cellular homeostasis and tumorigenesis, emphasizing the need for an integrated approach to understanding the *RAS*/Ras complex regulatory mechanisms and their implications for cancer treatment.

Understanding the conserved structure and domain-specific functions of Ras proteins lays the foundation for exploring how specific mutations convert *RAS* into potent oncogenes. These mutations do not simply disrupt isolated residues; they rewire intracellular signaling, interfere with protein–protein interactions, and, ultimately, enable uncontrolled cell growth and proliferation. Building on this foundation, the following section examines the molecular mechanisms through which *RAS* acquired transforming properties and drives tumorigenesis in both experimental models and human cancers.

## 3. RAS Transforming Properties: From Genes to Proteins

**Figure 2:**
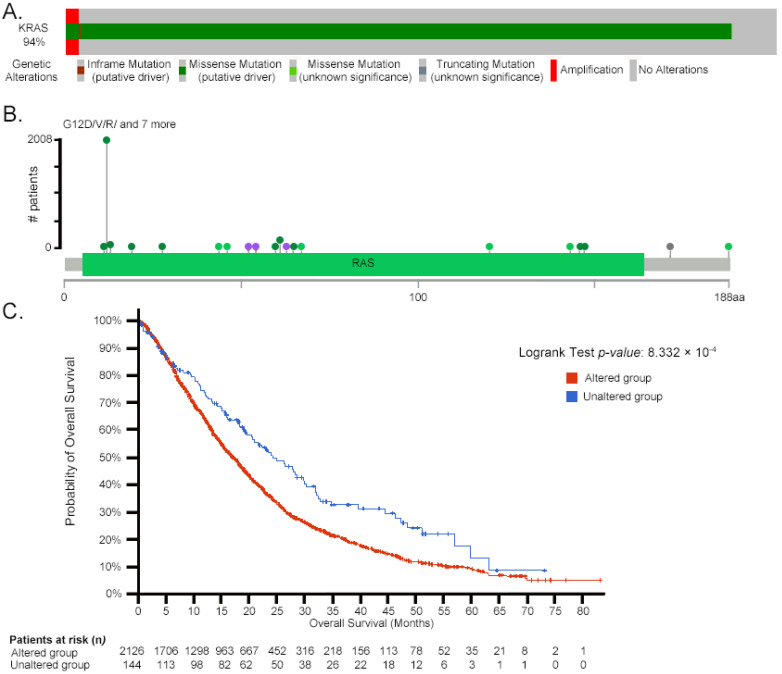
Correlation between *KRAS* mutations and patient survival in pancreatic adenocarcinoma. A: Oncoprint plot summarizing *KRAS* genetic alterations across patients. The top horizontal bar represents the number of patients with each alteration type, with each segment corresponding to one patient categorized by their mutation(s). Patients are visually grouped by mutation type in the following order: inframe mutations (purple), missense mutations—putative driver (dark green), missense mutations—unknown significance (light green), truncating mutations (gray), amplifications (red), and unaltered cases (light gray). B: The lollipop plot maps the distribution and frequency of mutations along the KRas protein sequence (188 amino acids), with each colored dot representing a specific mutation event: dark green (missense—putative driver), light green (missense—unknown significance), purple (inframe mutation), and gray (truncating mutation—unknown significance). The thin green line beneath the lollipops represents the mutation frequency per amino acid. aa: amino acid. C: Kaplan–Meier curve comparing overall survival in patients with KRAS-altered tumors (red curve) versus KRAS-unaltered tumors (blue curve). The x-axis represents survival time (months), and the y-axis shows the probability of overall survival. The number of patients at risk at each time point is listed below the x-axis. Log-rank test *p*-value: 8.332 × 10^−4^. Data adapted from cBioPortal [94,95].

Initially identified through point mutations, mammalian (human and mouse) *RAS/ras* can acquire transformation-inducing properties, leading to uncontrolled cell proliferation. Figure 2 illustrates the correlation between *KRAS* mutations and patient survival rates across select cancer types, based on publicly available datasets from cBioPortal [94,95], emphasizing the clinical impact of specific mutation hotspots such as glycine 12 (G12) and alanine 59 (A59).

Mutations at Gly12 are frequent and can substitute glycine with residues like arginine, valine, or aspartate. Depending on mutational context and selective pressure, alanine 59 can be replaced by threonine, serine, or other residues. These substitutions disrupt normal signaling because glycine and alanine are structurally crucial for Ras function [94,95]. For example, the Ala59→Thr substitution induces intramolecular autophosphorylation at a phosphate receptor site, although its in vivo functional relevance remains unclear [96,97].

Mutations at amino acid positions 63 [81,84], 116 [98], and 119 [84] have also been shown to confer transforming properties to Ras in various studies. Base substitutions at codons 12, 13, and 61, often triggered by exposure to carcinogens such as nitrosamines, polycyclic aromatic hydrocarbons, or radiation, activate Ras through mechanisms like methylation or acetylation in animal models [99,100,101]. In non-small-cell lung cancer, KRas mutations frequently occur at codons Gly12, Gly13, and Gln61, leading to constitutive activation of the protein and contributing to oncogenesis [102].

*RAS* mutations drive proliferative chronic myelomonocytic leukemia through the lysine methyltransferase 2A (KMT2A)–Polo-like kinase 1 (PLK1) axis [103]. PLK1 transcript levels regulate KMT2A activity by enhancing the monomethylation of lysine 4 on the promoter of histone 3, thereby influencing leukemogenesis. Similarly, loss of the transcription factor GATA-binding factor 2 (GATA2) reduces the viability of newly diagnosed non-small-cell lung cancer (NSCLC) cells harboring *KRAS* mutations, which drive aberrant KRas signaling [104]. In contrast, wild-type NSCLC cells remain unaffected.

Nuclear factor kappa B (NF-κB) also regulates abnormal cell proliferation and tumorigenicity in response to mutated *HRAS* [105]. In some contexts, NF-κB inactivation suppresses growth rather than inducing cell death following HRas activation. Additionally, NF-κB can drive cell proliferation and transformation by blocking apoptotic signals triggered by *RAS* mutations, a role distinct from its widely recognized anti-apoptotic function. NF-κB’s effects are highly context-dependent, varying by cell type and condition [87,106].

**Figure 3:**
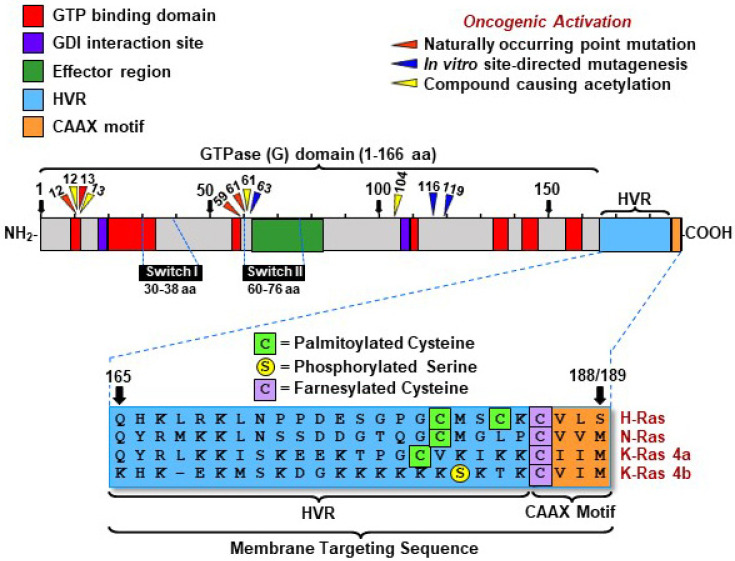
Structural and functional domains of mammalian RAS genes and Ras proteins. This diagram shows the major regions of mammalian *RAS genes* and their encoded *Ras proteins.* The G-domain (GTP-binding domain) governs molecular switching, while the hypervariable region (HVR) and CAAX motif enable membrane attachment. Downward triangles indicate amino acid positions commonly mutated in cancer, which are key targets for drug design. Abbreviations: GTP, guanosine triphosphate; GDI, guanosine dissociation inhibitor; HVR, hypervariable region; CAAX motif, a membrane-targeting signal consisting of cysteine (C), two aliphatic residues (A1, A2), and one variable amino acid (X). Numbers above triangles indicate residue positions.

The primary, secondary, and tertiary structures of Ras in mammals are well characterized, along with the partial characterization of its functional motifs [2,76,87,107]. The three proto-oncogenes of the *RAS* family, *KRAS*, *HRAS*, and *NRAS,* encode four highly homologous protein isoforms: HRas, NRas, Kras4a, and KRas4b [7]. Historically, approximately 85% of oncogenic mutations in *HRAS*, *KRAS*, and *NRAS* have been shown to contribute to tumorigenesis [108]. *NRAS* mutations are frequently observed in melanomas and certain hematopoietic malignancies, while *HRAS* mutations are prevalent in head and neck cancers [109]. In contrast, *KRAS* mutations are strongly associated with aggressive malignancies, occurring in 91% of pancreatic, 42% of colon, and 33% of lung cancers [8,70,110]. Figure 3 illustrates the structural and functional domains of mammalian *RAS* and Ras, emphasizing their role in tumorigenesis. In a transgenic mouse model, the mutated oncogenic *KRAS*^G12D^ caused the upregulation of solute carrier 25A1 (SLC25A1) by the glioma-associated oncogene 1 (GLI1) transcription factor. A high-fat diet also stimulated the K*RAS*^G12D^ → GLI1 → SLC25A1 pathway with the corresponding increases in citrate and fatty acids, promoting the development of pancreatic tumorigenesis [111].

KRas mutations at codon 61 (Q61) represent the least frequent hotspot for KRas, accounting for only 2% of KRas mutations in all cancers and 5% in PDAC [112]. These mutations involve substitutions such as Q61E (glutamine to glutamic acid), Q61H (glutamine to histidine), Q61L (glutamine to leucine), Q61P (glutamine to proline), and Q61R (glutamine to arginine). Among these, Q61H is the most common KRas Q61 mutation, occurring in 57% of cases, whereas Q61R is predominant in NRas and HRas, found in 47% and 43% of cases, respectively [113].

While the experimental models used to investigate these mutations (NIH 3T3 and RIE-1 cells) are not human derived, the study focuses on human KRas Q61 mutations and their oncogenic effects, particularly in PDAC [113]. These cell lines serve as tools for examining mutation-driven changes in a controlled setting, reinforcing their relevance to human biology. The functional impact of Q61 mutations is primarily determined by their biological activity, influencing cellular metabolism, signaling, and morphology [113]. While some information has been elucidated about the effects of amino acid-changing mutations, synonymous mutations are often non-neutral, and even silent mutations at codon 61 may pose therapeutic challenges in cancer treatment [114,115].

Six single-base missense mutations have been identified at Q61, with varying frequencies among cancer types [116]. Table 2 summarizes these mutations, highlighting Q61H as the most frequent (57%), followed by Q61R, Q61L, and Q61K, which account for 40%. The rare Q61P and Q61E mutations account for only 2% and 1% of cases, respectively [113]. Table 2 summarizes the different KRas Q61 mutations, their cellular effects, and their frequencies.

This section examines the critical role of feedback mechanisms and inter-protein interactions in driving Ras-mediated cell transformation. From a systems biology perspective, where interconnected components within a biological network collectively influence processes and outcomes, these transformations arise from complex signaling networks. Rather than halting tumor progression, disrupting a single component often perturbs the entire network and promotes adaptive resistance. Recognizing Ras as part of these feedback-driven networks reinforces the need for therapeutic strategies that target isolated mutations while addressing the dynamic pathways that sustain tumor growth and resistance.

While these mutations highlight the structural and signaling vulnerabilities exploited by cancer cells, the function of Ras extends beyond its role in oncogenic signaling. Its integration with metabolic pathways, particularly insulin signaling and glucose uptake, reveals how Ras proteins serve as nodal points between growth control and cellular energetics. The following section explores this metabolic interface, emphasizing Ras’ roles in modulating the Warburg effect and coordinating tumor proliferation with altered nutrient flux.

## 4. RAS and Insulin Receptor

In tumors, glycolysis is typically upregulated, characterized by an increased rate of glucose entry into cells—a phenomenon associated with the Warburg effect [41]. Although this metabolic reprogramming supports the biosynthetic and energetic needs of rapidly dividing cells, it remains unclear whether it is a driving force or a downstream adaptation in cancer progression. Warburg metabolism also decreases the likelihood of apoptosis by inducing mitochondrial changes, reducing reactive oxygen species, providing carbon skeletons for biosynthesis, and enabling tumor adaptation through lactic acid production and secretion [117]. Insulin, a critical regulator of glucose uptake, binds to its receptor on the cell membrane and triggers a cascade of intracellular events, including receptor autophosphorylation [118]. The activated insulin receptor phosphorylates downstream signaling intermediates, including adaptor proteins, G proteins, and Ras, initiating intracellular signaling cascades that enhance glucose transport into the cell [119]. Ras integrate signals from the insulin receptor, linking glucose metabolism with cellular proliferation and survival pathways, reflecting their dual function in metabolic regulation and signal transduction [120,121,122,123].

Insulin-induced activation of Ras has been implicated as an intermediary in the pathway linking receptor activation to gene expression [124]. When insulin binds to its receptor, it triggers a cascade that activates the RasGTP complex, selectively enhancing gene expression programs involved in glucose metabolism and growth regulation [120]. Overexpression or constitutive activation of Ras (particularly KRas) in insulin-responsive cells does not replicate all insulin-mediated effects, indicating that Ras selectively modulates specific branches of the insulin signaling pathway, primarily those associated with mitogenic responses [125].

Aberrant Ras signaling plays a significant role in metabolic reprogramming in both insulin resistance, a hallmark of type 2 diabetes, and cancer-associated metabolic dysregulation [126]. Hyperactivation of Ras signaling contributes to tumorigenesis by interfering with insulin receptor signaling, disrupting glucose uptake and metabolic homeostasis in affected cells [127]. *KRAS* is central in metabolic regulation in pancreatic cancer models, while *HRAS* and *NRAS* exhibit distinct functions in other cancer types [53,128]. These isoforms have differential signaling activities, influencing glucose metabolism depending on tumor type and mutation status [121]. This interference arises from the persistent activation of downstream pathways such as phosphoinositide 3-kinase (PI3K)–protein kinase B (AKT) and mitogen-activated protein kinase (MAPK), which are critical for insulin-mediated glucose metabolism [129]. Moreover, elevated Ras activity is associated with oxidative stress and inflammation, further exacerbating insulin resistance and creating a tumor-promoting microenvironment [130]. The systemic effects of Ras-driven metabolic alterations extend beyond cancer cells, impacting overall metabolic health. For instance, insulin resistance in adipose and liver tissues correlates with elevated circulating levels of insulin and glucose, which indirectly fuels tumor progression [131]. These findings underscore the intricate relationship between Ras hyperactivation, disrupted metabolic pathways, and the therapeutic challenges associated with targeting these processes.

Active Ras function as initiators rather than only effectors in insulin-mediated activities. Insulin stimulation increases Ras GTPase activity, which drives transcriptional programs involved in glucose metabolism [132,133]. For instance, the initial interaction between the insulin receptor and its ligand, followed by sustained Ras activation, facilitates continuous and enhanced glucose uptake, reflecting the dynamic interplay between Ras signaling and insulin pathways [118]. Ras signaling closely mimics insulin’s role in promoting glucose transporter translocation and uptake, highlighting its pivotal role in metabolic homeostasis. However, this pathway becomes hyperactivated in cancer cells, promoting a glycolytic phenotype that supports tumor growth and progression [134]. For example, Ras-driven activation of glucose transporters enhances metabolic flexibility in cancer cells, enabling them to survive under metabolic stress and evade therapeutic interventions [121].

Ras-driven metabolic reprogramming extends beyond glucose metabolism to encompass lipid biosynthesis and redox balance, pathways that allow cancer cells to sustain their rapid proliferation [131]. These adaptations underscore the versatility of Ras signaling in metabolic regulation, with implications that extend beyond individual cells to systemic metabolic health [131]. Dysregulation of these pathways is associated with increased therapy resistance in cancers with hyperactive Ras signaling, such as pancreatic, colon, and lung cancers [135]. Targeting this axis presents a potential therapeutic strategy to exploit the metabolic vulnerabilities of cancer cells while disrupting key oncogenic signals. Hyperactive Ras signaling has also been implicated in the development of insulin resistance within tumors. This resistance parallels systemic insulin resistance observed in obesity and type 2 diabetes, suggesting potential overlap in the mechanisms driving these conditions [126]. Such findings underscore the dual impact of Ras mutations on both systemic and tumor-specific metabolic regulation.

**Figure 4:**
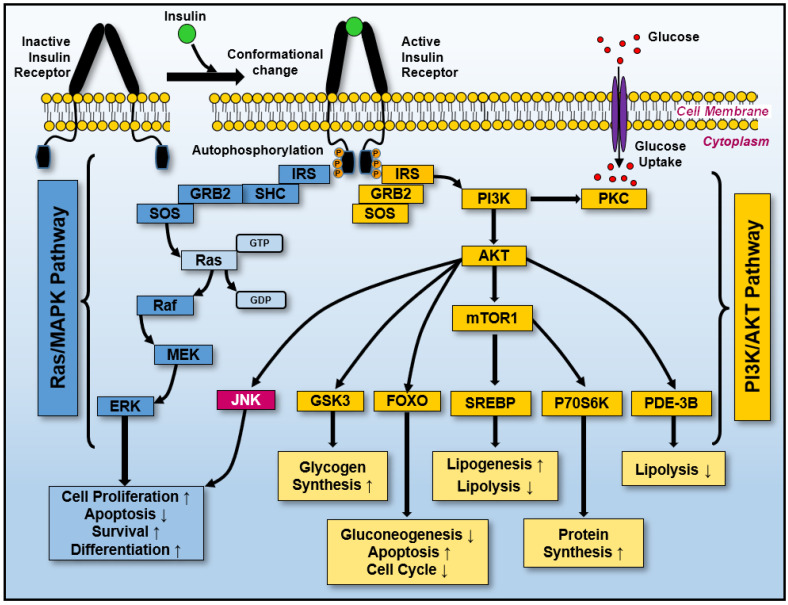
Activating insulin-induced receptors initiates two major signaling pathways: Ras/MAPK and PI3K/AKT. After insulin binds to its receptor on the cell surface, it triggers sequential structural conformational changes and autophosphorylation on several tyrosine residues localized in the cytoplasmic domains, eliciting its activation. These events recruit and phosphorylate the insulin receptor substrate (IRS), Src homology, and collagen (SHC) signaling proteins. The IRC-SHC’s complex activation primarily initiates the GRB2-SOS-Ras-MEK-ERK cascade, Ras/MAPK pathway (blue boxes), which elicits cellular proliferation, antiapoptosis, survival, and differentiation. Additionally, the IRS/GRB2/SOS activated complex induces PI3K-AKT-GSK3, PI3K–AKT-FOXO, PI3K-AKT-mTOR1-SREBP, PI3K-AKT-mTOR1-P70S6K, and PI3K-AKT-PDE-3B activation, all of which are components of the PI3K-AKT pathway (dark yellow boxes). The multiple cellular consequences of activating the PI3K/AKT pathway are included at the bottom of each pathway branch (yellow boxes). Moreover, protein kinase C (PKC) activation by PI3K can promote glucose uptake by inducing the translocation of glucose transporters to the plasma membrane. Several interconnections exist between these two pathways, such as JNK activation (highlighted in the red box). The orange “P” denotes phosphorylation (phosphate group addition). Note: ↑ and ↓ indicate increases (induction) and decreases (repression), respectively.

The interaction between Ras and insulin signaling is critical in maintaining metabolic homeostasis under normal and pathological conditions. Understanding this interplay provides insights into how cancer cells hijack homeostatic pathways to gain a growth advantage (Figure 4). Targeting the Ras–insulin axis has been explored as a strategy to restore metabolic balance, potentially increasing tumor cell susceptibility to therapeutic interventions [136]. This section emphasizes Ras as part of a broader regulatory network that governs metabolic pathways. Ras’ interaction with the insulin receptor is crucial for cellular homeostasis, and disruptions in this signaling cascade can trigger widespread metabolic dysregulation. The insights emphasize the importance of addressing Ras within interconnected cellular metabolism networks.

Beyond the canonical Ras/MAPK and PI3K/AKT pathways activated by insulin, Ras interacts with other molecular systems that influence metabolism and cell cycle progression. These non-canonical interactions, including those involving phospholipase enzymes and stable cell cycle regulators, reveal how Ras proteins coordinate proliferative and metabolic responses at multiple regulatory nodes. The following section explores these additional protein interactions, further illustrating Ras’ integrative role in maintaining cellular function under physiological and pathological conditions.

## 5. Other Ras Protein Interactions

Another aspect related to Ras’ interaction with insulin signaling involves phospholipase enzymes. Phospholipase C-mediated phospholipid degradation and phospholipase D activity are essential for the maturation of *Xenopus* oocytes—immature egg cells—in which Ras signaling plays a critical regulatory role during meiotic progression and insulin-driven cellular responses [137,138]. This includes increased phospholipid degradation in response to both Ras and insulin, although the precise mechanism by which Ras activates phospholipase C-mediated hydrolysis remains unclear. The level of Ras remains constant during the cell cycle while exerting functional effects. Although Ras levels remained steady in Xenopus oocytes, its GTP-bound active form triggered high M-phase-promoting factor activity, leading to rapid meiotic maturation [139]. This maturation suggests that while Ras quantity remains stable, it can still play a regulatory role during M-phase progression. Similarly, the cyclin-dependent kinase CDK1 (historically referred to as p34^CDC28^) maintains stable expression throughout the cell cycle yet remains metabolically active and essential for progression through mitosis [140]. These observations indicate that Ras does not require fluctuations in expression levels to exert regulatory functions. Additionally, all Ras interact with the same G protein as the insulin receptors, facilitating GDP-to-GTP exchange and further integrating metabolic and proliferative signaling.

Ras can remain active due to structural constraints within the G protein, kinase regulation, or a combination thereof [141,142]. Transformed cells, which have undergone genetic or epigenetic alterations leading to uncontrolled growth, exhibit a disruption in kinase activity, including abnormal activation or inhibition of specific kinases. This dysregulation is linked to increased inositol phosphate levels and a 2- to 2.5-fold accumulation of 1,2-diacylglycerol. In contrast, untransformed cells retain normal regulatory mechanisms and do not exhibit uncontrolled proliferation [143]. These alterations suggest a broader dysregulation of lipid signaling in *HRAS*-transformed cells.

Ras also influences additional layers of cellular regulation, including protein kinase C (PKC). Elevated PKC levels correlate with increased Ras expression [144,145], and cells with PKC overexpression or hyperactivity are more susceptible to transformation by *RAS* expression [146,147]. The accumulation of 1,2-diacylglycerol plays a dual role—initially activating, then downregulating PKC—by triggering its intracellular translocation and altering its access to substrates within downstream signaling cascades [143]. Because PKC can phosphorylate Ras effectors and modulate the Raf–MEK–ERK axis, its mislocalization in transformed cells may intensify aberrant Ras-driven proliferation and survival.

While phosphatidylinositol signaling is Ras-dependent rather than PKC-dependent [148], *HRAS*-transformed cells show 35% to 50% higher activity of phosphatidylinositol and phosphatidylinositol 4-phosphate kinases compared to untransformed controls, alongside a 25% to 30% reduction in 1,2-diacylglycerol kinase activity [143]. In addition, elevated inositol phosphate levels inhibit GAP activity on Ras, sustaining prolonged HRas signaling [149,150].

Quiescent cat embryo fibroblasts—non-dividing, metabolically inactive cells—require both the functional protein kinase C (PKC) pathway and the serum response element (SRE) pathway for Ras-mediated activation [151]. The SRE pathway regulates gene transcription linked to growth and proliferation, suggesting that Ras activation triggers multiple transcriptional programs during cell cycle re-entry. This dual-pathway activation underscores the integrative role of Ras in coordinating metabolic and transcriptional responses to stimulate cell proliferation. Cell transformation by Ras is associated with the persistent relocation of PKC from the cytosol to the cytoplasmic membrane—a shift that enhances substrate accessibility and alters downstream signaling dynamics [149]. Diacylglycerol, generated from phosphatidylcholine breakdown, contributes to this atypical regulation of PKC. Furthermore, Ras activates Na+/H+ channels through a PKC-independent mechanism [150]. Despite this, overall cellular osmolarity remains stable; tumorigenic cells do not become hypertonic (which would cause water loss) or hypotonic (which would lead to cell swelling from water influx).

Ras expression has been linked to broader biochemical metabolism, particularly calcium mobilization and activity [152,153,154]. Calcium is required for protein kinase C activity, and 1,2-diacylglycerol enhances its affinity for calcium. However, an experiment examining calcium’s role in cell metabolism influenced by Ras found no net increase in intracellular calcium [155]. One explanation is that intracellular calcium becomes sequestered by binding proteins or organelles, such as the endoplasmic reticulum (ER), preventing the formation of free calcium pools despite increased mobilization during tumorigenesis [156,157]. This suggests that while Ras-driven signaling promotes calcium flux, most calcium remains unavailable for downstream signaling [158]. Thus, calcium mobilization desensitization arises either from partial inhibition of inositol 1,4,5-trisphosphate, which regulates the calcium channel, or from HRas interfering with calcium translocation among intracellular components.

Another mechanism underlying calcium regulation in membrane potential involves protein kinase signaling. While this discussion focuses on protein kinase B (PKB/Akt), other kinases may also contribute. For instance, deficiencies in the phosphatidylinositol 3-kinase-protein kinase B/Akt (PI3K-PKB/Akt) pathway are associated with endocrine tumors, highlighting its crucial role in carcinogenesis [159]. Additionally, the activation of the Src family tyrosine kinase/phosphoinositide 3-kinase/protein kinase B (Lyn/PI3K/Akt) and mitogen-activated protein kinase/extracellular signal-regulated kinase (MAPK/ERK) pathways—both of which intersect with Ras effector cascades—confers cellular protection under oxidative stress [160]. This crosstalk between Ras signaling and oxidative stress response may contribute to tumor cell survival in hostile microenvironments. Simultaneous activation of phosphatidylinositol 4,5-bisphosphate 3-kinase catalytic subunit alpha/Akt (PIK3CA/AKT) pathway and *RAS* mutations suppress Ras-induced senescence, a state of permanent cell cycle arrest that limits proliferation but retains metabolic activity and may promote inflammatory factor secretion [161]. Furthermore, these findings support the notion that increased Ras-driven tumorigenesis can occur without activating the PIK3CA/AKT pathway in vivo. In human tumors, hypoxia-inducible factor-1 (HIF-1), an oxygen-dependent transcriptional activator normally degraded under normoxic (normal oxygen) conditions, is upregulated due to intratumoral hypoxia. Under normoxic conditions, prolyl hydroxylase domain (PHD) proteins hydroxylate HIF-1α, marking it for proteasomal degradation. However, in hypoxic environments, PHD activity is suppressed, allowing HIF-1α to accumulate and dimerize with HIF-1β to form the active transcription factor [162]. *RAS* mutations induce HIF-1α785 (a HIF-1 isoform) overexpression through the Raf/mitogen-activated protein kinase/ERK kinase (MEK)/extracellular-signal-regulated kinase (Raf/MEK/ERK) pathway. As a result, the splice variant HIF-1α785 plays a crucial role in *RAS*-driven tumor promotion by trans-activating approximately 60 target genes [163]. Ras activator-like 2 (RASLA2) is a negative regulator of Ras signaling, particularly relevant in tumors with high *RAS* mutation frequencies [4]. In PC-3 prostate cancer cells—a widely used cell line derived from human prostate adenocarcinoma—RASAL2 overexpression suppresses cell proliferation and metastasis by downregulating Ras expression [4].

Ras activates adenylate cyclase [164]. Mutational analysis identified a region on adenylate cyclase that interacts with and sequesters Ras. Adenylate cyclase functions through two distinct G protein transducing systems that respond to stimulatory and inhibitory receptors. Through interactions with G protein-coupled receptors and downstream signaling pathways, Ras indirectly modulates adenylate cyclase activity by affecting G protein activation and subunit dynamics [165]. In *Drosophila*, ras signaling is essential for proper embryonic and neural development; loss-of-function mutations lead to defects in eye formation, wing vein patterning, and central nervous system differentiation. The rutabaga gene encodes a Ca^2+^/calmodulin-responsive adenylate cyclase, and Ras signaling has been shown to regulate its activity in *Drosophila*, linking Ras to cAMP dynamics in the mushroom bodies—the centers for olfactory learning and memory in flies [166]. Mutations in rutabaga impair olfactory learning and memory, underscoring the importance of Ras-regulated cAMP signaling in these processes [166].

While *rutabaga* is not cancer-related, the conserved Ras–adenylate cyclase-cAMP axis underscores Ras’ broader role in housekeeping regulation and oncogenic signaling. Additionally, Ras-mediated modulation of adenylate cyclase impacts synaptic plasticity at the *Drosophila* neuromuscular junction, reinforcing Ras’ broader role in neural signaling [167]. These comparative models highlight the conserved yet distinct roles of Ras-mediated adenylate cyclase regulation in humans, mice, and *Drosophila*, reinforcing its significance in diverse physiological processes.

The phorbol ester 12-O-tetradecanoylphorbol-13-acetate enhances adenylate cyclase responsiveness, leading to elevated cAMP levels [139]. This suggests that PKC-mediated modulation of cAMP signaling may counteract Ras-driven oncogenic pathways, potentially by altering downstream effector activation or transcriptional responses. This effect may reflect cAMP’s ability to activate protein kinase A (PKA), inhibiting Raf-1, thereby dampening the Ras–Raf–MEK–ERK signaling cascade.

Moreover, cAMP inhibits the growth-related and transformation-specific properties of Ras [168]. Elevated cAMP levels in Ras-activated cells suggest an interaction between Ras and phosphodiesterase 4 [144], a cAMP-specific enzyme responsible for degrading intracellular cAMP pools. This interaction may form the basis for an intracellular delivery system for anti-Ras therapeutics. Such a system represents a promising strategy for developing novel Ras-targeted cancer therapies. For instance, a bioengineered chimeric toxin utilizing the diphtheria toxin translocation system fused with Ras-proximate-1 (Rap1) or Ras-related protein 1 (RRSP) could irreversibly downregulate Ras activity [169]. This chimeric RRSP–diphtheria toxin fusion (RRSP-DTᴮ) has been shown to cleave intracellular RAS irreversibly (both mutant and wild-type) at picomolar concentrations, halting downstream signaling and dramatically reducing tumor growth in breast, colon, and other xenograft models—without significant toxicity—serving as proof of concept for pan-Ras targeting in vivo [170]. Furthermore, this chimeric toxin provides proof of concept for RRSP as a potential pan-Ras inhibitor, a therapeutic agent that inhibits HRas, KRas, and NRas isoforms to prevent oncogenic signaling, demonstrating efficacy in both in vitro and in vivo models.

**Figure 5:**
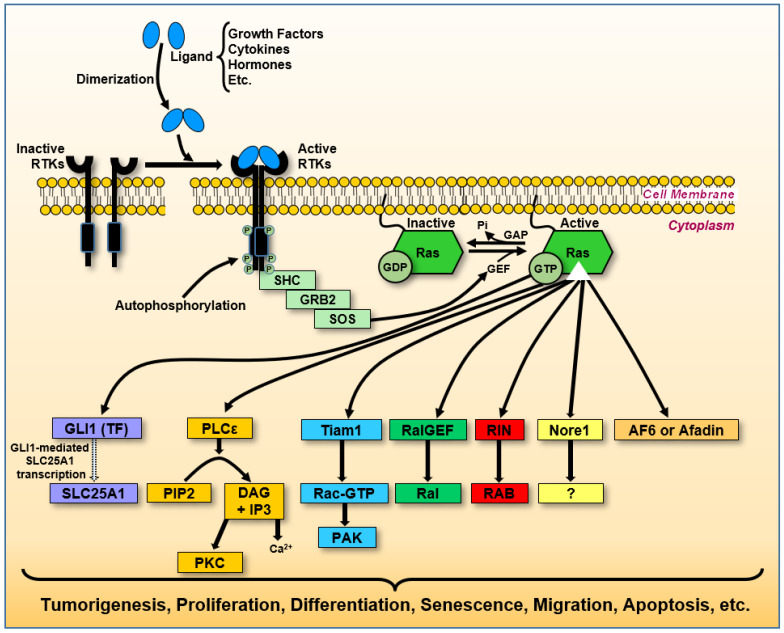
Ras signaling networks and their oncogenic impact. Upon the activation of receptor tyrosine kinases (RTKs), Ras initiates multiple signaling cascades that influence various cellular functions. Adaptor protein GRB2 binds SOS, a guanine nucleotide exchange factor (GEF), forming a complex that facilitates GDP–GTP exchange on Ras. This figure illustrates key pathways: Mutant KRas^G12D^ enhances SLC25A1 expression via the GLI1 transcription factor, promoting pancreatic tumorigenesis (purple boxes). In this context, the arrow from GLI1 to SLC25A1 reflects transcriptional activation, consistent with GLI1’s role as a nuclear transcription factor. Ras stimulates phospholipase C epsilon (PLCε), converting phosphatidylcholine inositol 4,5-bisphosphate (PIP2) into second messengers diacylglycerol (DAG) and inositol 1,4,5-trisphosphate (IP3), leading to intracellular Ca^2+^ release and activation of protein kinase C (PKC), which regulates secretion, ion conductance, gene expression, and cell proliferation (yellow boxes). Ras activates Tiam1, modulating Rac1 activity, which subsequently activates PAK1, influencing actin cytoskeletal reorganization, cell survival, proliferation, and motility (indicated by light blue boxes). Ras interacts with RalGEF and Ral proteins, regulating cell growth and survival, which has implications for cancer development (green boxes). Ras associates with RIN and RAB GTPases, regulating protein transport along endocytic and exocytic pathways (red boxes). Upon Ras activation, Nore1 may induce cell cycle arrest and apoptosis, suppressing tumor cell growth. Ras activates Afadin, facilitating the formation of adherent junctions during embryogenesis and modulating signal transduction, migration, invasion, and apoptosis in cancer progression (bright orange box).

Framing these interactions within a system-level perspective reveals how Ras dynamically interacts with molecular complexes that adjust in response to cellular changes. This adaptability exemplifies the complexity of cancer progression, particularly in advanced stages, where targeting individual proteins may be insufficient to achieve a complete cure. A systems-oriented approach to Ras signaling could inform more effective therapeutic strategies by accounting for the interconnected molecular networks that sustain tumor growth and resistance. These diverse interactions illustrate how Ras signaling integrates metabolic, proliferative, and structural pathways to regulate cellular function. Figure 5 summarizes the key Ras-mediated signaling cascades, highlighting their roles in oncogenesis, cytoskeletal organization, and intracellular signaling dynamics.

In addition to regulating intracellular signaling networks, Ras may influence biophysical properties at the cell membrane. Emerging evidence suggests that Ras activity can alter membrane potential, adding a new dimension to its role in cancer progression. The following section explores this under-recognized mechanism, examining how Ras-mediated electrical changes at the membrane interface could affect protein interactions, ion flux, and cell fate decisions.

## 6. Ras, Membrane Proteins, and Membrane Potential

The hypothesis that Ras disrupts membrane potential has been explored. As a contextual parallel, non-ionizing electromagnetic fields (EMFs) are known to induce calcium loss and destabilize membrane-associated proteins, occasionally leading to membrane disintegration [171]. Interestingly, carcinoma cells display oscillatory membrane hyperpolarization resembling the effects seen under EMF exposure [172]. While not Ras-specific, these observations suggest that external or internal disruptions to membrane potential can broadly influence membrane stability and protein localization. In direct support of Ras involvement, expression of Ras, particularly with a point mutation at codon 12, has been shown to depolarize the resting membrane potential and increase membrane conductance, the ease with which ions flow across the membrane, in NIH 3T3 fibroblasts [173]. These shifts correlate with suppressed proliferation of melanoma cells grown under standard in vitro conditions [174]. Changes in membrane potential regulate interactions between membrane-bound proteins and lipid-soluble molecules such as tocopherols and tocotrienols, which can modulate the activity of Ras, G proteins, or kinases, potentially leading to their inactivation. Thus, oscillations in membrane potential triggered by oncogenic Ras transformation may be a critical mechanism driving abnormal cellular growth.

Although the precise nature of Ras-mediated membrane potential changes remains incomplete, current data suggest a tight link between these electrical shifts and the spatial dynamics of membrane-associated proteins, especially those responsive to lipid composition or transmembrane voltage gradients.

The plasma membrane is a barrier and a dynamic platform for signal integration, ion flux regulation, and protein localization. Alterations in membrane potential and lipid composition have been documented in various transformed cells, including those expressing oncogenic Ras. For instance, NIH 3T3 fibroblasts transformed with HRas codon 12 mutations exhibit depolarized resting membrane potentials and increased sodium ion (Na^+^) permeability, changes that correlate with Ras activation and may reflect downstream bioelectric consequences of oncogenic transformation [175]. These shifts in membrane dynamics extend beyond ion gradients; they actively modulate the function and positioning of critical signaling proteins, including small GTPases, kinases, and phospholipid-binding domains. Accordingly, Ras proteins, through interactions with “lipid rafts”—cholesterol- and sphingolipid-rich microdomains that organize signaling molecules—and other membrane microdomains, may exert control over downstream effectors by altering the biophysical landscape of the membrane itself [176].

Additional evidence from non-Ras systems reinforces the plausibility that membrane dynamics influence protein localization—a principle increasingly relevant to Ras biology. For example, Annexin V, a calcium-dependent phosphatidylserine-binding protein, which translocates from the inner to the outer leaflet of the plasma membrane during apoptosis in response to phosphatidylserine exposure [177]. While classically associated with cell death, Annexin V illustrates a broader mechanism by which membrane charge and lipid asymmetry can drive spatial repositioning of proteins. In Ras-transformed cells, similar membrane remodeling may contribute to the relocalization of key regulatory factors, including PEBP1 (RKIP), G protein subunits, and phosphoinositide-binding effectors. Notably, the downregulation of PEBP1 in Ras-transformed cells [178] may reflect transcriptional repression and a functional displacement from its inhibitory targets due to altered membrane properties.

Furthermore, oncogenic Ras disrupts membrane lipid asymmetry and clustering, including within detergent-resistant membrane microdomains, also known as lipid rafts, which serve as hubs for the assembly of signaling complexes [176]. Such disruptions may not only affect canonical signaling cascades, such as the Raf/MEK/ERK pathway, but also influence the localization and activity of lipid-metabolizing enzymes like phospholipase D and A2, which contribute to arachidonic acid release and subsequent prostaglandin production [179,180]. These lipid messengers themselves can feed back into Ras regulation. Lipid-derived prostaglandins such as PGF2α and PGA2 have been shown to stimulate RasGAP activity, whereas prostacyclin (PGI_2_) inhibits it, suggesting a feedback mechanism through which lipid metabolism influences Ras signaling fidelity [180].

**Figure 6:**
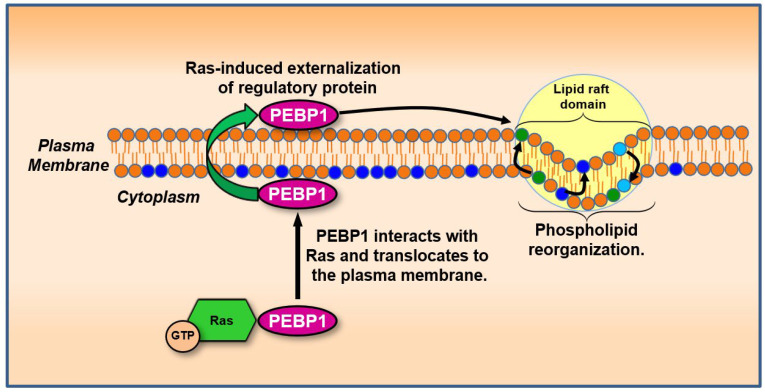
Membrane protein relocalization driven by Ras-induced lipid and electrical changes. Activation of Ras alters membrane potential and lipid microdomain composition, disrupting or enabling specific protein–membrane interactions. This figure shows how phospholipid-binding proteins such as phosphatidylethanolamine-binding protein 1 (PEBP1) can translocate from the cytosol to the plasma membrane in response to these biophysical changes. Proteins in this class, including Annexin-like molecules, may be guided by lipid dynamics and electric gradients, influencing pathways involved in mitogenic signaling or suppression. GTP: guanosine triphosphate. Color coding of the lipid bilayer: Orange circles represent general phospholipids that form the bilayer. Dark blue circles indicate negatively charged phospholipids (e.g., phosphatidylserine), light blue circles denote neutral phospholipids (e.g., phosphatidylethanolamine), and green circles mark positively charged or zwitterionic lipid interactions critical to domain stability and protein targeting. Black arrows within the bilayer indicate Ras-driven reorganization and translocation processes.

Altogether, this evidence supports a model in which Ras-induced changes in membrane potential and lipid architecture shape a dynamic membrane environment that influences the localization of signaling proteins. Whether these shifts mimic apoptosis-associated movements, such as those of Annexin V, or generate new protein distributions unique to transformation, they underscore a potential interplay between membrane biophysics and Ras signaling dynamics. Recognizing this interplay may be key to understanding Ras function in tumorigenesis and identifying points of intervention where membrane-associated signaling becomes therapeutically targetable. Further study into protein–membrane interactions in Ras-transformed cells may uncover novel biomarkers and regulators of oncogenic growth (Figure 6).

Biochemical data further support the role of Ras in membrane potential regulation [181]. Ras exerts allosteric control through the modulation of arachidonate metabolism [182]. Arachidonic acid is a key fatty acid precursor to eicosanoid hormones, including prostaglandins and thromboxanes, influencing various cellular processes. Using recombinant HRas and RasGAP, along with neurofibromin, the protein product of the NF1 gene, and the catalytic GAP domains, prostaglandins PGF2α and PGA2 stimulate RasGAP activity, whereas prostacyclin (PGI_2_) inhibits RasGAP function. Although these prostaglandins were not directly activated, arachidonic acid inhibited neurofibromin’s catalytic activity, suggesting a regulatory interaction between arachidonic acid metabolism and Ras signaling.

Neutral lipid domains within the plasma membrane can undergo structural reorganization in response to signaling inputs, altering membrane permeability and protein recruitment [183]. Such changes in membrane lipid composition have been linked to shifts in biological function, including ion channel activity and signal transduction [184]. In the context of Ras biology, KRas has been proposed to act like a biological transistor—responding to upstream signals, such as lipid microdomain changes or receptor activation, to modulate membrane potential and selectively gate downstream signaling pathways [181]. However, the precise role of lipid domains and membrane potential in Ras-mediated processes such as cell–cell recognition, transformation, and tumorigenesis remain unresolved. Further investigation is needed to determine how Ras interacts with other membrane regulators within this signaling landscape, including ion transporters and phosphoinositide-binding proteins.

In mammals, phosphatidylethanolamine-binding proteins (PEBPs), an evolutionarily conserved protein family, are associated with membranes and phospholipids [185,186,187,188]. Studies investigating *RAS*-driven cellular transformation have reported reduced levels of PEBP1 [189], a protein later shown to inhibit Raf1 kinase activity and proposed to function as a metastasis suppressor based on its ability to restrain mitogenic signaling [190]. The discovery that PEBP1 inhibits Raf1, a central component of the Raf/MEK/ERK signaling pathway, became recognized as a Raf kinase inhibitory protein (RKIP). The Ras/RAF/MAPK cascade transmits extracellular cues to the nucleus to regulate cell growth, division, and differentiation. This pathway also plays essential roles in wound healing, tissue repair, integrin signaling, and cell migration [191]. Additionally, it stimulates angiogenesis by modulating gene expression to facilitate the formation of new blood vessels [192]. As such, Ras/RAF/MAPK signaling is a central regulator of tumorigenesis. In addition to Raf1, PEBP1 regulates other protein kinases such as GRK2, NF-κB-inducing kinase (NIK), and IκB kinase (IKK), primarily by disrupting protein–protein interactions or phosphorylation events within their respective signaling pathways [178,193]. Another protein binding to this enhancer region, PEBP2, which plays a role in mammalian spermatogenesis and post-testicular maturation [194], undergoes conversion to PEBP3 under conditions favoring dephosphorylation. This PEBP2-to-PEBP3 conversion has been observed in Ras-transformed cells and may contribute to sustained mitogenic signaling associated with tumorigenesis. These findings highlight the need for further investigation into the role of membrane dynamics in distinguishing usual cellular suppressor functions from cancerous processes, even when both mechanisms operate concurrently.

**Figure 7:**
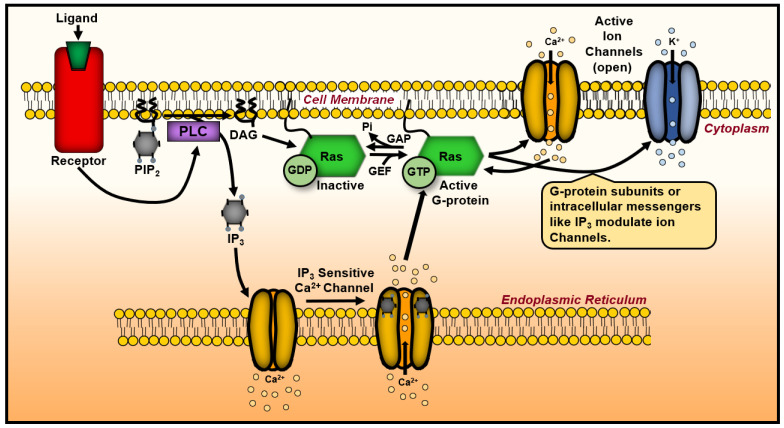
A schematic representation of Ras’ influence on ion channels and membrane potential. Upon activation, Ras associate with the plasma membrane, modulating ion channels, thereby altering ion flux and membrane potential. Ras-induced changes in membrane lipid composition reorganize membrane microdomains, including lipid rafts, which affect downstream signaling cascades. These events create feedback mechanisms that regulate Ras activity and broader cellular functions. DAG, diacylglycerol; GAP, GTPase-activating protein; GEF, guanine nucleotide exchange factor; IP_3_, inositol 1,3,5-trisphosphate; PIP_2_, phosphoinositide; PLC, phospholipase C; GTP, guanosine triphosphate.

This section explains how Ras influences and is influenced by membrane potential within a broader cellular context. Changes in membrane potential impact entire signaling networks, highlighting the interconnectedness of membrane dynamics with cellular signaling pathways (Figure 7). This interconnected perspective underscores the importance of considering cellular events as integral to a cohesive system, where alterations in one factor, such as membrane potential, can propagate widespread effects on cell behavior, growth regulation, and tumorigenesis.

While membrane potential modulation reflects a biophysical mechanism of Ras influence, its evolutionary origins point to a very different source of disruption: microbial and viral interactions. The discovery that *RAS* genes share similarities with viral oncogenes suggests a deeper connection between cellular transformation and the ancient integration of retroviruses. The following section explores this relationship, tracing how viruses may have shaped the *RAS* family’s regulatory logic and oncogenic potential.

## 7. Ras and Microbe Connection

In 1970, researchers studying retroviruses, viruses capable of integrating into the host genome, confirmed the existence of oncogenes, a class of genes that can drive cancer development [195]. Later, the *RAS* family was identified as a proto-oncogene in mammalian systems. They discovered that various mechanisms could activate or mutate these genes, leading to uncontrolled cell proliferation.

Many oncogenes have been traced back to retroviruses that acquired host DNA fragments from previous infections. This process led to the hypothesis that proto-oncogenes originated from ancient retroviruses, which incorporated genetic material from earlier host infections into their viral genomes. Over time, these viral sequences became integrated into the host’s germline DNA and were inherited through generations. These viral remnants, known as endogenous retroviruses, persist in the genome and may contribute to oncogenic transformation under certain conditions.

Tracing the evolutionary history of oncogenes like *RAS* can reveal conserved molecular features and vulnerabilities that targeted cancer therapies may exploit. This perspective also underscores the role of ancient viral integrations, such as endogenous retroviruses, in shaping oncogenic pathways. Studying the mechanisms that activate or mutate proto-oncogenes into oncogenes provides insights into novel cancer prevention and treatment strategies. The discovery of these retroviral elements has broad implications for understanding the genetic basis of cancer and the emergence of virally derived oncogenes, including *RAS* [8,70,111,196]. These oncogenic drivers illustrate how microbial elements can shape tumor evolution, contributing to the selective pressures that drive mutation-specific oncogenic pathways. These insights highlight the significance of examining microbial influences in cancer inhibition and therapeutic resistance, where viral and bacterial interactions with Ras-driven tumors may impact drug efficacy and disease progression.

Two studies have shown that adenoviral DNA tends to be unmethylated when chromosomally integrated and actively expressed, whereas silenced viral DNA is heavily methylated [197]. Similarly, in herpes saimiri-transformed cells, methylated episomal viral DNA corresponds with the absence of detectable viral production [198]. These viral examples highlight how methylation can regulate gene expression. In the context of Ras, aberrant methylation patterns, whether hypermethylation of regulatory regions or hypomethylation of integrated sequences, may contribute to inappropriate Ras expression. Such epigenetic modulation may act alongside mutational events, reflecting how microbial elements influence Ras-driven oncogenesis through insertional mutagenesis and disruption of host epigenetic control.

Ancient viral elements may still influence Ras biology today. The Human Genome Project revealed that while roughly 8% of the human genome consists of ancient retroviral remnants, up to 40% may be composed of repetitive retroviral sequences [199]. These ancient integrated retroviruses are known as human endogenous retroviruses [200]. These human endogenous retroviruses (HERVs) are typically transcriptionally silent but retain the potential to influence gene regulation. Although oncogenic retroviruses are classified as acute or slowly transforming, these two frameworks may not fully account for long-term oncogenic influences. A third conceptual category may be warranted: endogenized viral sequences whose latent regulatory elements can modulate oncogenesis across generations. In the context of Ras biology, this raises a provocative possibility: that under certain conditions, such as inflammation, epigenetic reprogramming, or microbial co-infection, HERV elements may become reactivated, altering the transcriptional environment surrounding *RAS* or its regulators. If true, this mechanism would represent a long-overlooked layer of Ras pathway modulation, driven not by mutations alone but by latent viral architecture embedded in the human genome.

Colorectal cancer-associated genetic alterations, including *KRAS* mutations, interact with the microbial community, either shaping its composition or being influenced by microbial activity [201]. Similarly, microbial interactions have been linked to oncogenic signaling beyond colorectal cancer. NRas activation by microbial components has been implicated in pathogen-induced proinflammatory responses [202]. Additionally, bacterial toxins from *Clostridium difficile* and *Clostridium sordellii* have been shown to modify Ras GTPases through glucosylation, suggesting a potential mechanism by which microbial toxins drive inflammatory responses [203]. Beyond these interactions, Ras proteins also play broader roles in host–microbe dynamics. In the amoeba *Dictyostelium discoideum*, Ras and Rac coordinate cytoskeletal remodeling during phagocytosis, a process crucial for engulfing extracellular material [204]. This process involves Rac, a small Ras GTPase, and RGBARG, a multidomain protein containing RCC1, RhoGEF, BAR, and RasGAP domains. However, the specific proteins facilitating this organization remain unidentified in mammalian cells.

**Figure 8:**
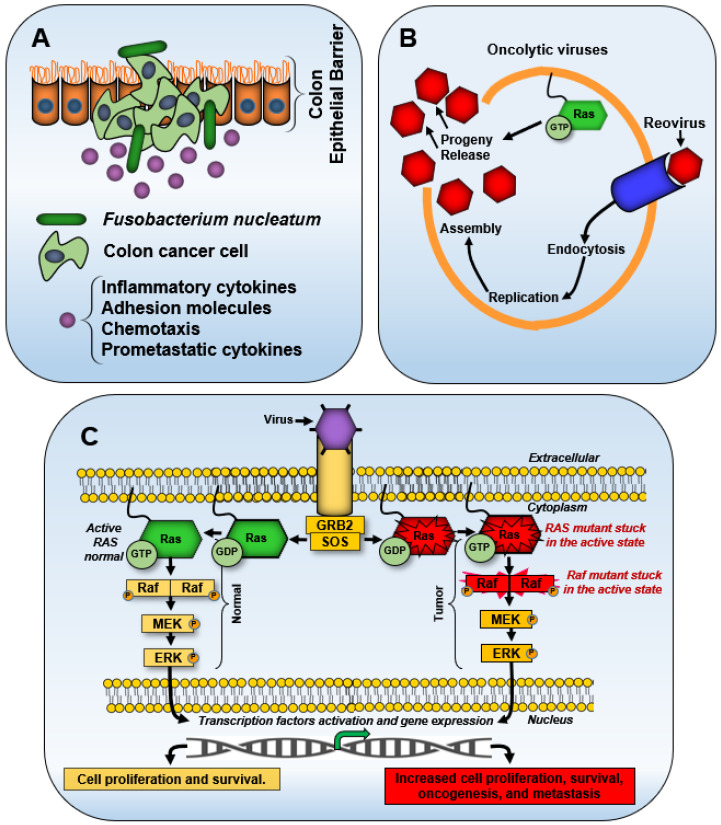
Mechanisms of general microbial modulation of Ras signaling. (**A**) *Fusobacterium nucleatum* is associated with *KRAS* mutation, reprogramming the tumor microenvironment by recruiting tumor-infiltrating immune cells and releasing pro-metastatic cytokines. (**B**) Reovirus is a virus that can replicate in cells with activated Ras signaling, promoting the release of progeny virions and oncolysis. (**C**) Adenovirus and hepatitis B virus (HBV) alter cell cycle progression by activating the Ras/RAF/MEK/ERK pathway, driving uncontrolled proliferation, oncogenesis, and metastasis.

*Fusobacterium nucleatum* (Fn) bacteria are a topic of investigation in colorectal cancer (CRC). Fn, typically found in the oral cavity, is significantly more abundant in CRC tissues than in healthy colorectal tissues (Figure 8A) [205]. Fn intestinal colonization may be a risk factor for KRAS-mutant CRC and the sessile serrated neoplasia pathway [206]. Fn favors CRC by adhering, invading, and inducing inflammatory responses via cytokine production in the cellular colon epithelium, overstimulating the proliferation of colorectal cells and supporting progression to oncogenesis and metastasis [206].

In human cancers such as colorectal, liver, and lymphoid malignancies, the myeloid differentiation factor 88 (MyD88) protein, an adaptor in innate immune signaling, links inflammatory responses to bacterial and viral infections with Ras-driven signaling [207]. Microbial associations with *KRAS* mutations have been identified, with *Bacteroides* emerging as the genus most strongly correlated with *KRAS*-mutant tumors. Fourteen distinct microbes have been linked to *KRAS* mutations, underscoring potential microbial contributions to oncogenesis [208]. Clinical studies have consistently detected Fn, a bacterium strongly associated with colon cancer, in colon tumor samples [209]. Its presence in resected tumor tissues supports a causative role in colorectal cancer rather than being a mere byproduct of tumor growth [210].

Reovirus, a virus capable of selectively infecting and lysing cancer cells, preferentially replicates in Ras-transformed cells to enhance virion disassembly, viral progeny production, and viral release through apoptosis. When Ras is active, oncolytic viruses infect and destroy cancer cells by promoting the release of progeny. Due to this feature, they are considered a potential anticancer treatment (Figure 8B) [211].

To further illustrate this interplay, four viral examples, adenovirus type 2, HBV, reovirus, and the human immunodeficiency virus type-1 (HIV-1), demonstrate how Ras contributes to the oncogenic potential of exogenous viral factors. The N-terminal half of adenovirus type 2 represses innate immune responses and forces host cells into S-phase, creating a permissive environment for viral replication. It also modulates Ras signaling by enhancing the activity of downstream effectors such as ERK, thereby promoting cell proliferation and immune evasion [212]. Similarly, HBV is implicated in hepatocarcinogenesis in chronically infected patients by activating the Ras/RAF/MAPK pathway, which drives hepatocyte transformation (Figure 8C) [213]. Reovirus, a virus capable of selectively infecting and lysing cancer cells, preferentially replicates in Ras-transformed cells by exploiting enhanced Ras signaling to promote virion disassembly, viral protein synthesis, and progeny release via apoptosis [214]. The virus also alters Ras localization within the Golgi apparatus, a change that enhances apoptosis and increases viral release, mechanisms that may be exploited in oncolytic virotherapy targeting Ras-driven tumors.

Regarding HIV-1, Ras signaling contributes to the accumulation of amyloid-beta in the brain through interactions with the HIV-1 trans-activator of transcription (Tat) protein [215]. Additionally, human herpesvirus 6 (HHV-6) has been shown to suppress cellular transformation driven by HRAS overexpression, while concurrently inhibiting HIV-1 replication by downregulating transcription from the virus’s long terminal repeat (LTR) promoter region [216]. This interplay between contemporary viruses, endogenous retroviral proteins, and oncogenic processes demonstrates how microbial interactions, both ancient and modern, shaped cellular homeostasis and tumorigenesis.

These microbial and viral interactions with Ras reflect deep evolutionary pressures and adaptive responses that shape normal cell function and disease. Viewed through an evolutionary lens, they offer an integrative perspective on Ras roles in health and oncogenesis. A systems-level approach reveals that Ras–microbe interactions form part of a broader dynamic network that influences cellular outcomes, from tumor suppression to tumor promotion, depending on context. These findings show how viruses and bacteria can exploit Ras-driven signaling and potentially shape long-term oncogenic trajectories. As such, they blur the boundary between external environmental insults and internal regulatory mechanisms, setting the stage for concluding insights into Ras biology and its role in cancer progression.

## 8. Conclusions

**Figure 9:**
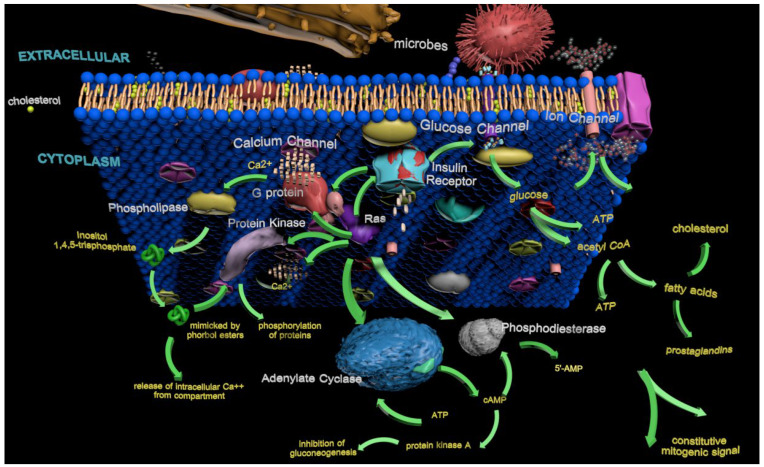
Three-dimensional highlight of the complex roles of Ras in general housekeeping functions and tumorigenesis. This figure illustrates the multifaceted interactions of Ras within the membrane (lipid bilayer), cytoplasm, and extracellular environment, including microbes. Ras influences intracellular signaling pathways, metabolic networks, and external stimuli, integrating biochemical cues that regulate normal cellular homeostasis and tumorigenic transformation. The diagram maps key molecular players and interactions that shape cellular growth, differentiation, immune response, and energy dynamics. These processes involve ATP production, G protein signaling, and other mechanisms, as well as microbial influences, all of which contribute to the delicate balance between physiological function and oncogenesis.

Reviewing *RAS* and Ras, from intracellular signaling networks to membrane-associated functions, may offer insights and a deeper understanding of their roles in the pathogenesis of late-stage human cancers while preserving essential housekeeping functions (Figure 9). This knowledge could also inform strategies to counteract first-generation retroviral exposure before viral integration into the genome leads to nth-generation retroviral infections, where descendants may inherit integrated viral sequences that contribute to cancer susceptibility.

Humans already carry remnants of ancient viruses within their genomes, acquired through viral integration events that occurred eons ago. Similarly, today’s exogenous retroviruses, such as HIV-1, could, over evolutionary time, become endogenous, potentially adopting cellular housekeeping roles in future generations. Understanding this process could aid in developing preventative strategies against retroviral-driven oncogenesis, particularly for individuals at high risk of chronic retroviral exposure.

Even though the *KRAS*^G12C^ mutant is an oncogenic driver in multiple cancer types, it has long been considered “undruggable”. However, recent advances in small-molecule inhibitors targeting KRas mutant proteins have shown promising progress in clinical trials [217]. In *KRAS*^G12C^ mutations, a glycine-to-cysteine substitution at position 12 accounts for approximately 44% of all *KRAS* mutations [218].

Among these advances, Sotorasib (AMG510) is a first-in-class inhibitor of KRas^G12C^ mutants, approved in the United States and the European Union. Sotorasib binds irreversibly to KRas^G12C^, inhibiting downstream signaling pathways related to cell proliferation and differentiation [219]. It is currently recommended as a therapy for adults with advanced non-small-cell lung cancer (NSCLC) carrying the *KRAS*^G12C^ mutation, particularly in patients who have previously undergone platinum-based chemotherapy and/or immunotherapy [219]. Sotorasib has exhibited a 37.1% overall response ratio in NSCLC patients following at least one prior line of therapy [220].

Specific allosteric inhibitors for KRas^G12C^, which involve glycine-to-cysteine mutations at residue 12, demonstrate clinical activity in cancer patients [221]. Adagrasib (MRTX849) has emerged as another U.S. Food and Drug Administration-approved covalent inhibitor targeting KRas^G12C^ with promising drug-like properties. Adagrasib has demonstrated marked tumor regression in 65% (17 of 26) of KRas^G12C^-positive cell lines and patient-derived xenograft mouse models representing various tumor types, including lung and colon adenocarcinomas [32]. Two clinical trials demonstrated that combining Adagrasib with pembrolizumab (an inhibitor of lymphocyte PD-1 receptors) is a safe and effective approach in patients carrying a *KRAS*^G12C^ mutation with newly diagnosed non-small-cell lung cancer (NSCLC) [222,223]. The response rate was 49% and 57% in the two trials. The drug combination also exhibited lower levels of liver toxicity compared with alternative inhibitor-based and targeted therapy regimens. Adagrasib is also used in advanced NSCLC patients carrying *KRAS*^G12C^ mutations. Adagrasib is used in patients with advanced non-small-cell lung cancer (NSCLC) carrying KRASG12C mutations. A previous clinical study of this population achieved a 45% overall response rate [224].

Most patients, however, do not respond favorably to KRas^G12C^ inhibitor therapy due to intrinsic or acquired resistance driven by cellular, molecular, and genetic mechanisms [217]. In contrast, no direct inhibitors are available for HRas, NRas, or non-G12C KRas variants [221]. However, a potent, non-covalent, and selective KRas^G12D^ (glycine-to-aspartic acid mutation at residue 12) inhibitor, MRTX1133, was recently discovered [225]. After intraperitoneal administration, it successfully treated xenograft mouse tumors with the *KRAS*^G12D^ mutation. Moreover, an MRT1133-derived prodrug for oral administration has also been identified, demonstrating effective antitumor activity in a xenograft mouse tumor model expressing the KRas^G12D^ mutant [226].

Understanding the proto-oncogenes and oncogenes of *RAS* and the associated Ras has evolved significantly over the decades, deepening insights into their roles in cancer and cellular signaling. Key *RAS*/Ras research milestones were identified through targeted PubMed searches, selecting highly cited and influential articles representative of each decade from the 1980s to the present. These selected references highlight foundational discoveries, significant conceptual advances, and therapeutic developments relevant to RAS biology and cancer. From the 1980s to the present, scientific advancements have continuously reshaped our knowledge of *RAS*/Ras functions [2,84].

In the 1980s, discovering *RAS* as an oncogene marked a pivotal moment in cancer research, emphasizing how mutations transform *RAS* from a common gene into a tumorigenic driver [64,227]. By the early 1990s, identifying Ras as a GTPase and its role in signal transduction pathways laid the foundation for understanding its impact on cell growth and differentiation [85,228]. The late 1990s highlighted the mitogen-activated protein kinase (MAPK) pathway, emphasizing Ras’ critical function in cell division and cancer progression [229,230]. This period also introduced knockout mouse models as vital tools for studying *ras* functions [231].

In the 2000s, research shifted toward targeted therapies, focusing on *KRAS*, the most prevalent *RAS* variant in pancreatic cancer [54,232,233]. As the decade progressed, *RAS* gained recognition as a biomarker for cancer prognosis and treatment response, while epigenetic modifications of *RAS* emerged as an area of interest [234].

The 2010s introduced challenges related to resistance to Ras-targeted therapies and led to the exploration of synthetic lethality as a therapeutic strategy [235,236]. The advent of CRISPR/Cas9 technology in the mid-2010s provided new possibilities for directly editing *RAS* [237]. Concurrently, liquid biopsies emerged as a noninvasive method for detecting *RAS* mutations [238].

Building on these foundations, research has increasingly focused on developing pan-Ras inhibitors and advancing precision medicine, tailoring treatment plans based on a patient’s *RAS* status [31,239]. These advancements underscore the dynamic nature of RAS/Ras research and its continued relevance in the urgent fight against cancer. We refer to reviews that compile this evolving data for readers interested in detailed outcomes from ongoing trials involving Adagrasib, Sotorasib, and emerging inhibitors [240,241,242,243].

The observations presented in this work provide a foundation for understanding the complex roles of *RAS* and Ras in housekeeping functions and tumorigenesis. These roles raise a critical question: What is still missing in explaining the challenges associated with late-stage human cancers? For instance, further exploration of the biological mechanisms underlying the effects of Ras on membrane potential and the biochemical detection of Ras conformational changes under human physiological conditions may advance therapeutic strategies. Fluidics-based experimental platforms, such as microfluidic and millifluidic systems, may offer more physiologically relevant methods for studying Ras behavior and drug response [244,245,246,247,248,249]. These approaches move beyond the limitations of conventional models like the MCF-7 cell line or the C57BL/6J (B6/J) mouse, which are often selected based on availability, cost, or routine use rather than biological relevance to human tumors.

Current late-stage cancer treatment strategies remain inconsistent, often rooted in outdated paradigms that fail to capture the complexity of tumor evolution and therapeutic resistance [1]. A major driver of poor outcomes is the inherent heterogeneity of tumors: even when treatments eliminate 99.9% of cancer cells, the remaining 0.1%, often genetically and epigenetically distinct, can expand rapidly, repopulating the tumor and driving relapses [245,250,251]. Some researchers have proposed that cancer is fundamentally a mitochondrial metabolic disease, dependent primarily on oxidative phosphorylation (OXPHOS) for survival. However, this view is increasingly seen as incomplete. Emerging research shows that tumor metabolism is highly plastic, influenced by treatment exposure, local environment, and cellular state. Cancers shift between glycolysis and OXPHOS based on these conditions [252,253,254]. This adaptability challenges the notion of a universal metabolic model and raises concerns about the relevance of in vitro findings. Expanding research into physiologically relevant models, rather than relying solely on conventional cell lines, may offer more accurate insights into the behavior of late-stage human cancers.

This work emphasizes the need to revisit how RAS and Ras are conceptualized in cancer biology. While many studies treat RAS/Ras mutations as discrete targets, this reductionist view often overlooks how these proteins operate within broader signaling networks that integrate metabolic cues, immune responses, and stress adaptation. By reframing RAS/Ras as dynamic hubs within a multi-layered system, rather than isolated drivers, we can better understand why targeted therapies often fail in late-stage cancers, especially when faced with cellular plasticity and feedback mechanisms. A systems-level approach is not simply a conceptual stance but a necessary shift toward therapeutic designs that anticipate complexity, resistance, and tumor heterogeneity from the outset.

## Figures and Tables

**Table 1:** Percentages of patients with some cancer types carrying *KRAS* gene mutations.

Cancer Type	% of Patients Carrying *Kirsten RAS* (*KRAS*) Gene Mutation *	Reference
Pancreatic	90–92	[13]
Thyroid	41.3–52.6	[14]
Colon	30–50	[15]
Colorectal	35–45	[16]
Lung	25–50	[17]
Myeloid Leukemia	10–30	[18]

* The percentages can change over time.

**Table 2:** The summary of the types of KRAS mutation focusing on the substituting glutamine residue at position 61, their cellular effect, and frequency in cancers.

Type	Mutation at Glutamine Residue 61 to	Cellular Effect	Frequency (%)
KRAS^Q61H^	Histidine	Disrupted actin cytoskeletal organization	57
KRAS^Q61K^	Lysine	Inhibits both GAP and intrinsic GTP hydrolysis	40 Collectively for the three mutations.
KRAS^Q61L^	Leucine	Disrupted actin cytoskeletal organization	
KRAS^Q61R^	Arginine	Disrupted actin cytoskeletal organization	
KRAS^Q61P^	Proline	Disrupted actin cytoskeletal organization	2
KRAS^Q61E^	Glutamic acid	Stimulated actin stress fiber formation	1

## Data Availability

Not applicable.
